# The Role of Physical Frailty Independent Components on Increased Disabilities in Institutionalized Older Women

**Published:** 2019-01-06

**Authors:** GE Furtado, R Letieri, A Caldo, M Patricio, M Loureiro, E Hogervorst, JP Ferreira, AM Teixeira

**Affiliations:** 1 Research Unit of Physical activity and Sport at Faculty of Sport Sciences and Physical Education (CIDAF; UID/PDT/04213/2019) – University of Coimbra, Portugal; 2 Laboratory of Biostatistics and Medical Informatics and IBILI, Faculty of Medicine of the University of Coimbra; 3 School of Sport and Exercise Sciences, Loughborough University, United Kingdom

**Keywords:** Frail older adults, falls, disability evaluation, activities of daily living, motor skills

## Abstract

The purpose of this study was to identify the independent components of physical frailty that most influence disability indicators in institutionalized older women. A cross-sectional study with 319 participants (81.96±7.89 years old) was performed. Disability was assessed through dynamic and static balance tests, activities of daily life and falls risk screen. Fried physical frailty protocol was used to access physical frailty. The frail subgroup displayed the weakest results for all disability indicators (p < 0.05). Regression analysis showed that in the two models tested, low physical activity levels and slowness were the physical frailty independent components that better associated with the disability indicators. More studies with larger samples will help to better understand the independent relationship of each physical frailty component with disability outcomes and assist to design a co-adjuvant treatment to reverse physical frailty.

## I. INTRODUCTION

The Frailty Syndrome is a state in which reserve function across multiple physiologic domains decline, compromising the individuals’ capacity to withstand stress. This situation predisposes them to poor general health, functional decline, institutionalization and death [1], [2]. Fried et al.[3] developed a protocol to identify Physical Frailty (PF) that evaluated five components: weakness (low grip strength), low resistance to effort (exhaustion), slowness (poor gait speed), low physical activity levels, and (non-intentional) weight loss, and categorized the population in frail, pre-frail and non-frail subjects [3].

Recent findings suggest that PF is strongly linked to different Functional Disability (FD) indicators, leading to increased caregiver burden, and greater financial costs for public health[4]–[6]. Currently, FD is understood as a multidimensional construct that integrates the analysis of compensation strategies to maintain a satisfactory physical health and perform daily life activities autonomously[7]. The assessment of FD can be quantified directly (through simple and low cost functional fitness and motor tasks tests) or indirectly (through questionnaires evaluating specific daily life tasks), both clinically validated [8], [9]. Positive changes in FD affect quality of life and perception of a positive physical condition (e.g. not being afraid to face possible physical barriers that condition the fear of falling) might reflect a personal sense of predicted support from others, despite being physically frail.

Currently, there is poor information about the contribution of each PF independent component on physical-functional decline [10]. Epidemiological studies reported that poor scores on the handgrip strength test were associated with high risk of mortality [11], dementia, and mild cognitive impairment [12]. Low levels of physical activity were consistently associated with mortality [13], cognitive decline [14], poor walking speed proficiency, and low physiological reserve [15]. Geriatric researchers have started to explore the independent association of each PF component with some indicators of FD [6]. However, the relationship between the PF independent components, different FD indicators and their contribution to understand the functional decline of frail institutionalized-dwelling individuals, is still poorly explored. Thus, the aim of this study was to analyze the relationship between the PF independent components and FD indicators and to explore the differences on functional disability between the different physically frail subgroups in institutionalized-dwelling older women.

## II. METHODOLOGY

This is a cross-sectional study design that included institutionalized-dwelling older adults over 75 years old. Five centers of health care and social support (CHS) were approached and agreed to participate in this study.

### Sample selection criteria

Participant inclusion and exclusion criteria were validated through face-to-face interview. Participants were excluded when severe chronic illnesses (e.g. severe cardiomyopathy, hypertension, uncontrolled asthmatic bronchitis), any musculoskeletal impairments that could prevent performance of the physical tests to assess functional disability, mental disorders (e.g. Alzheimer and Parkinson, severe dementia), severe hearing or vision impairment, morbid obesity, having no controlled and updated drug therapy or the use of medications that could cause attentional impairments and disturb the motor activity (e.g. anxiolytics and anti-depressants) were present.

### Participants and sample size statistical power

Initially, 483 institutionalized-dwelling participants were approached to participate in this study. However, 164 women were excluded due to a poor clinical health condition (with high physical and cognitive impairment). A total of 319 older women were finally included in the study. Statistical power was computed by considering the Lawton index values to compare differences between frail and pre-frail groups, using a Mann–Whitney U test, with a significance level of p = 0.01. The analysis was performed on G*power 3.1.9.2; the power was determined to be 0.97 with a sample of 300 and an effect size of 1.12 [16].

### Ethical procedures

All the CHS directors and potential subjects who expressed interest in participating in the study signed an informed consent form, in which the privacy and anonymous identity of the data collected were guaranteed, and any needed access to the participants medical records was given. This study protocol was approved by the University of Coimbra Faculty of Sport Sciences and Physical Activity, Ethical Committee (reference code: CE/FCDEF-UC/000202013) respecting the current guidelines for human research of the Helsinki Declaration [17].

### Data collection

Data collection was performed by the principal investigator and by a trained research team constituted by a nurse, physiotherapist and kinesiologist. The following variables were assessed: sociodemographic information, anthropometry, global health status (clinical-mental health status), physical frailty (unintentional weight loss, exhaustion, weakness, slowness, and physical activity level), and functional disability outcomes. To minimize inter-variability between evaluators, only one researcher was responsible to collect data from all participants in this study. The quality data of physical tests (static balance, agility-dynamic balance, hand grip and 15-feet walking tests) was examined in the pilot data collection and internal consistency reliability (ICR) for each test was reported.

### Sociodemographic screening

Chronological age was assessed through the date of birth and analyzed as a continuous variable. Marital status was categorized as single, married, widowed or divorced. Level of education, assessed as a continuous variable, was collected for each participant and classified according to the Portuguese educational system [18].

### Anthropometric measures

The standardized procedures described by Lohan and colleagues were followed for the collection of anthropometric data [19]. To assess body mass, a portable scale (Seca®, model 770, Germany) with a precision of 0.1 kilograms was used. For stature, a portable stadiometer (Seca Body meter®, model 208, Germany) with a precision of 0.1 centimeters was used. Participants, body mass index (BMI) was calculated according to the standard formula (BMI = body mass/stature^2^).

### Clinical and mental health status

Comorbidity was assessed using the Charlson Comorbidity Index (CCI) that measures the burden of disease and has a weighted index based on 19 comorbid conditions. The score ranging from 0 (low) to 10 points (high) combined with age and gender provides a single index [20]. Individuals presenting a comorbidity index above 10 points were excluded from the study. The Mini-Mental State Examination (MMSE) questionnaire was used to assess cognition; the maximum score is 30 points and a score below 24 is usually considered indicative for dementia screening [21], [22]. Depression was assessed using the scale developed by the Centre of Epidemiologic Studies in Depression (CES-D)[23]. The 20-items scale has an overall score ranging from 0 to 60 points where the highest scores correlate with the frequency of depressive symptoms [24].

### Physical Frailty screening

The incidence of PF was calculated based on a continuous score ranging from 0 to 5 points of the following five components: unintentional weight loss, exhaustion, weakness, slowness, and physical activity level. Unintentional weight loss was assessed by self-reporting a loss of four kilograms or more in the last six months, validated by medical records over one year. Exhaustion (self-reported weariness) was evaluated by negative concordance of questions number 7 and 20 from the CES-D scale [23]. Weakness was analyzed using the handgrip strength test (HGT). This test uses a hand-held dynamometer (Lafayette Dynamometer, model 78010, United States) and strength is measured in kilograms. The subject holds the dynamometer in the hand to be tested, with the elbow by the side of the body. When ready, the subject squeezes the dynamometer with maximum isometric effort, which is maintained for 5 seconds. The best result of the two trials was used for scoring purposes [25]. Participants who were unable to perform the HGT and those in the lowest 20% (adjusted by gender and BMI) were categorized as positive, based on cut off values of Fried’s study population; Slowness was measured using the 4.6 meters walking test (4.6-WT) which results are expressed in seconds and adjusted for gender and stature. Based on cut off values of Fried’s study population, the best time of the two trials was used for final scoring [15]. Physical activity (PA) levels were assessed by the International Physical Activity Questionnaire (IPAQ) short version [26]. The IPAQ short form asks about three specific types of activity undertaken and time being sedentary. The types of activity assessed were walking, moderate-intensity activities and vigorous intensity activities. Frequency (measured in days per week) and duration (time per day) are collected separately for each specific type of activity. The total volume and the number of day/sessions were included in the IPAQ analysis. There are four levels of PA suggested for classification: inactive, minimally active, medium active and a highly active. Participants classified as inactive and minimally active had a positive score for the frailty status [27].

According to this five components, participants were categorized as pre-fail (scored positively in one or two PF components), frail (scored positively in three or more components), and non-frail (scored negatively in all five frailty independent components) [3].

### Functional disability indicators

The assessment of FD was organized in a protocol proposed in a previous study [7]. The Katz Index of Independence (ADL) and the Instrumental Lawton Index (IADL) were used to assess autonomy in daily life tasks. The ADL scale ranks adequacy of performance in six tasks (dressing, transferring, toileting, continence, feeding, and bathing). Individuals are scored for each function as independent (1 point) or dependent (0 points) for each task. A score of 6 indicates full function, 4 indicates moderate impairment, and 2 or less indicates severe functional impairment [8], [28]. The Lawton Instrumental Activities of Daily Living (IADL) was used to identify deterioration or improvement over time in 8 socio-biological functions. A summary score ranges from 8 to 32 points, low function (dependent) to high function (independent) [29]. Fear of falling was measured using the Tinetti Falls Efficacy Scale (FES), which individuals are asked to rate, on a 4-point Likert scale, their concerns about the possibility of falling when performing 16 activities. Scores range from 10 to 100 points, with a lower score indicating a high self-efficacy and little fear of falling [30]. The eight foot-up-and go test was used to assess agility-dynamic balance (ADB). The ADB test measures the total time in seconds needed for the participant to get up from the chair, walk the distance of 2.44 meters as quickly as possible around either side of a cone, and to sit back down in the chair (ICR = 0.80). A total time of more than 9 seconds indicates a “risk zone”. [31]. The Tandem Stance Balance test (TSB) was used to evaluate static balance; it consists of the participant maintaining the standing position with eyes opened and one foot in front of the opposite foot for a maximum of 30 seconds, 10 seconds or less indicating very poor static balance (ICR = 0.77). Both TSB and ADB tests were chosen for their easy in application and clinical validity in older populations [32]. Three repetitions of each physical test were performed and the best score was considered.

### Statistical analysis

The Shapiro-Wilk test was used to assess normality of continuous variables. Normally distributed continuous data was described by their averages and standard deviations. Non-normally distributed continuous data was described by median and first and third quartiles. As for categorical variables, absolute and relative frequencies were used. Independent samples ANOVA tests and Kruskal-Wallis tests were performed to compare continuous variables between groups and Chi-square tests to assess the association between categorical variables. Spearman’s rank correlations and corresponding partial Spearman correlations were used to test the associations between FD outcomes and PF total score. The relationships between the Katz index of ADL and each of PF independent components was evaluated using logistic regressions. The relationships between PF and all other disabilities outcomes were analyzed using linear regressions. The unadjusted model simply included one dependent variable and one independent variable. In model 1, stature, comorbidities, depressive state, and cognitive state were included as covariates. The outcomes of disability were assumed as dependent variables and the PF components were independent in the regression analysis. The magnitude of the associations was classified as follow: trivial (r < 0.1), small (r = 0.1 to 0.3), moderate (r= 0.3 to 0.5), strong (r=0.5 to 0.7), and robust (r= 0.7 to 0.9) [33]. The level of significance adopted was 0.05. All computations were performed on IBM/SPSS Statistics 21 and R version 3.3.1.

## III. RESULTS

Characterization of the sample is shown in Table 1. Total sample mean age was 81.96±7.89 years, 68% of the participants were divorced or widowed, and the median level of education was third grade. When analysing the differences between the frailty subgroups, no significant changes were found for sociodemographic and anthropometric variables, except for stature. Mean stature showed that the frailest individuals had a significant shorter stature (p = 0.008). Frail individuals presented a lower score on the cognitive test (p < 0.001), a higher depressed mood (p = 0.026), and a higher comorbidity index (p < 0.001).

Functional disability outcomes showed significant differences for all variables, the frail subgroup being more dependent (Katz index of ADL = p < 0.001 and Lawton index of IADL p = 0.002), having higher fear of falling (p = 0.002), poorer static (p = 0.039) and dynamic balance (p < 0.001) when compared to pre-frail and non-frail subgroups (table 1).

Table 2 presents the Spearman’s rank and partial correlations controlling for covariates and shows the statistical differences in the group-treatment comparison. A moderate-to-strong correlation between the PF composed score and all the FD indicators was found (p<0.005). The results of partial correlations controlling for age, education, comorbidities and depression, showed that only the correlation between the Lawton index of IADL and the FES scale disappeared.

Multivariate regression analyses were performed and the results are presented in table 3. Two models of independent relationships were generated between each disability and frailty independent components. The unadjusted results showed that weakness was significantly associated with Katz of ADL and DBT tests, slowness was associated with all FD outcomes, exhaustion was associated with all FD components (except the STB test) and low PA levels were also associated with all FD outcomes, except for the FES scale. However, all these associations were trivial (r < 0.10).

After adding the adjusted covariates, weakness presented a significant trivial correlation with the Katz of IADL index (β = −0.047; OR = 0.954; IC95% [0.898, 1.016]; p <0.001) and ADB test (r^2 =^ 0.18; β = −0.325; p = 0.007). The PF component of slowness also showed a small correlation with the Katz of ADL index (β = 0.28; OR = 1.316; IC95% [1.076, 1.609]; p <0.001), a moderate association with the ADB test (r^2 =^ 0.53; β = 1.982; p <0.001) and a trivial correlation with the TSB test (r^2 =^ 0.05; β = −0.444; p = 0.034). Self-reported exhaustion maintained an independent and small significant association with the Lawton of IADL index (r^2 =^ 0.53; β = 1.982; p <0.001) and a significant but small association with the ADB test (r^2 =^ 0.21; β = −6.642; p = 0.001). The independent PF component of weight loss presented a small association with the Katz of IADL index (β = −1.707; OR = 0.181; IC95% [0.044, 0.749]; p < 0.001) and a trivial association with the TSB test (r^2 =^ 0.05; β = −4.379; p = 0.016).

Low PA levels had the smallest association with the Katz index (β = −0.245; OR = 0.783; IC95% [0.509, 1.204]; p <0.001), a moderate association with the Lawton index of IADL (r^2 =^ 0.30; β = −1.182; p = 0.010) and a small association with the ADB test (r^2 =^ 0.25; β = −3.528; p = 0.010). Lastly, residual and analytical analyses did not show violations of the assumptions underlying regression analysis and indicated a satisfactory fit of the model.

## IV. DISCUSSION

The aim of the present study was to investigate the relationship between PF and FD outcomes in institutionalized women over 75 years old. We also examined the disability differences between the three frail sub-groups. The main findings were that low levels of PA and slowness were the PF components most associated with FD outcomes, even after adjusting the models. On the other hand, the Katz index of ADL and the ADB test were de FD outcomes most associated with the physical frailty independent components in both regression models.

After Linda Fried described the PF Phenotype [3], several studies examined the prevalence of physical frailty in institutionalized samples [34]. Studies performed in Spain [35], [36], North America [37] and Brazil [38] corroborate the findings of the present study with similar prevalences of PF (45.4%), with the frail individuals having the worst FD scores when compared to the other PF subgroups.

The other finding that drew attention was the short stature found in the frail subgroup. According to previous evidence, stunted growth as a developmental delay is a risk factor for later life functional impairments [39], [40]. Stature could be related to osteopenia/osteoporosis leading to loss of height. This fact was independent of age and needs to be further explored. Previous researchers have also found that a high comorbidity index, lower cognition and depressive status also appear to strongly associate with physical frailty [41], [42].

As in the present study, low PA levels have been found to be a PF independent component with a relationship with the Katz of ADL and Lawton of IADL indexes [43]–[45]. The construct of daily life activities includes underlying socio-biological functions that are highly dependent on a satisfactory level of PA [46]. The biological mechanisms remain poorly comprehended, however, the main effects of low PA on functionality could be mediated by reduced muscle strength, and possibly by inflammation and a down-regulated sex steroids hormone expression [47].

Gait speed is the most used motor skill test in studies related to physical performance in older frail populations [48]. Low capacity of walking speed has been found to be an independent component of physical frailty linked with FD outcomes in numerous previous findings [49], [50]. On the other hand, the Katz of ADL Index was the FD outcome most closely related with all the independent components of frailty and may explain the physical deficit on the functional status that occurs in an advanced frailty stage. For this reason, some researchers recognize it as an independent maker of frailty status [51].

Additionally, the results showed that, not the static balance, but the dynamic balance motor skill test had the best relationship with all the independent components of the frailty status. This test has shown satisfactory associations with the PF independent components in previous studies [52], [53]. In addition, recent research has demonstrated that the ADB test is a good predictor of PF; and is used for instance, when the full application or interpretation of Fried’s criteria is impracticable [52], [54], [55]. A critical analysis of the ADB test can help understand the satisfactory associations with PF found in this study since this test requires the integration of different physical capacities such as time reaction, upper body strength and agility [56], [57]. Exploring these associations in institutionalized-dwelling individuals has a particular interest since their risk for age-physical decline was approximately four times higher when compared to community-dwelling individuals.

## V. CONCLUSION

Our findings showed that low levels of PA and slowness are the independent components of physical frailty most associated with functional disability outcomes. However, this study has some limitations that should be addressed: this is a cross-sectional study design, associations between the variables may be bidirectional; smaller number of people in the frailty subgroups due to excessive and unexpected number of dropouts

More epidemiological studies are needed, across different sample cohorts of institutionalized-dwelling populations, to determine the real prognostic value of frailty independent components and to help design a co-adjuvant treatment to prevent frailty based on active lifestyle police interventions, aiming to increase levels of PA and at encouraging changes in sedentary behaviors in this population.

## 







## Figures and Tables

**Table 1:** Characterization of total sample and comparison of physical frailty subgroups for biosocial, global health and functional disability outcomes

	Total sample (n=319, 100%)	Nonfrail (n=49, 16%)	Pre-frail (n=124, 38.7%)	Frail (n=146, 45.4%)	p value
	
**Sociodemographic**					
Chronological age (years, A±SD)	81,96 (±7,89)	81.68 (±6.72)	81.80 (± 8.65)	82.19 (± 7.72)	0.959
Level of education (degree; M1;3)	3 (3; 4)	4 (3; 6)	3 (3; 4)	3 (2; 4)	0.063
Marital state (n,%)					
Single	31 (26.1)	6 (31.6)	12 (26.1)	13 (24.1)	
Married	7 (5.9)	4 (21.0)	1 (2.2)	2 (3.7)	0.073
Widowed or divorced	81 (68.0)	9 (47.4)	33 (71.7)	39 (72.2)	
**Anthropometric data**					
Weight (kilograms, A±SD)	65.45(±12.58)	66.22 (± 11.33)	65.08 (±11.54)	65.49 (± 13.98)	0.946
Stature (meters, M1;3)	1.51 (1.47; 1.56)	1.56 (1.49; 1.62)	1.51 (1.47; 1.55)	1.50 (1.46; 1.52)	**0.008**
Body mass index (A±SD)	28.49 (± 5.05)	26.95 (± 3.78)	28.22 (± 4.60)	29.27 (± 5.69)	0.205
**Clinical-mental health state**					
Mini mental state (0–30 pts, M1;3)	20 (15; 25)	25 (21; 27)	21 (17; 25)	17 (13; 22)	**< 0.001**
Comorbidity index (0–10 pts, M1;3)	7 (6; 9)	8 (6; 10)	7 (6; 8)	8 (7; 9)	**0.026**
CES-D depression scale (0–60 pts, A±SD)	21.92 (± 8.00)	19.42 (± 7.99)	19.46 (± 8.09)	24.89 (± 6.98)	**0.001**
**Functional Disabilities indicators**					
Katz index of ADL (0–6 pts, n,%; no disability)	43 (36.1)	13 (68.4)	20 (43.5)	10 (18.5)	**< 0.001**
Katz index of ADL (0–6 pts, n,%; no disability)	76 (63.9)	6 (31.6)	26 (56.5)	44 (81.5)	
Lawton index of IADL index (9–32 pts A±SD)	20.11 (± 5.70)	17.37 (± 7.24)	18.70 (± 5.27)	22.28 (± 4.65)	**0.002**
Falls efficacy scale (10–100 pts M1;3)	40.00 (18.00; 61.00)	33.00 (14.00; 40.00)	34.50 (13; 70)	41.00 (26.00; 59.00)	**0.048**
Static balance test (per time, seconds M1;3)	1.30 (0.05; 4.11)	2.52 (0.71; 11.00)	1.56 (0.17; 4.15)	1.09 (0.01; 3.38)	**0.039**
Dynamic balance test (per time, seconds M1;3)	13.00 (10.00; 20.56)	9.75 (7.12; 10.58)	11.15 (9.20; 14.90)	20.14 (14.30; 25.97)	**< 0.001**

A=Average (mean), SD=standard deviation, M1; 3= Median (25^th^ Percentile; 75^th^ Percentile); pts = points

**Table 2:** Characterization of total sample and comparison of physical frailty subgroups for functional disability outcomes

	1	2	3	4	5
1. ADL					
2. IADL	0.513**				
	0.472**				
3. FES	0.176	0.436**			
	0.114	0.329**			
4. DBT	0.428**	0.387**	0.321**		
	0.347**	0.261**	0.231*		
5. SBT	−0.255**	−0.299**	−0.253*	−0.466**	
	−0.240**	−0.261**	−0.227*	−0.449**	
6. PF	0.420**	0.327**	0.247**	0.662**	−0.224*
	0.303**	0.149	0.140	0.610**	**−0.194**

Notes:

* p< 0.05 and

** p< 0.010;

in each variable line are expressed r and (p) values; partial correlation values are expressed in underline of each variable line and was adjusted for pre-determined covariates; PF =Physical frailty total score; ADL = Katz index; IADL = Lawton index; DBT = Dynamic balance test; SBT= static balance test.

**Table 3:** Association between each physical frailty and functional disability components (n = 319)

Functional Disability outcomes	Katz’s index of ADL				Lawton’s index of IADL			Falls efficacy scale			Agility-dynamic balance test			Static Balance test		
Physical Frailty components	β coefficient	OR	95% CI for OR	omnibus test p-value	R^2^	β coefficient	p value	R^2^	β coefficient	p value	R^2^	β coefficient	p value	R^2^	β coefficient	p value
**Low hand grip strength test (Weakness)**																
Unadjusted	−0.056	0.946	[0.898, 0.996]	**0.028**	0.016	−0.096	0.166	<0.001	0.069	0.825	0.095	−0.400	**0.001**	0.006	0.070	0.415
Adjusted*	−0.047	0.954	[0.896, 1.016]	**<0.001**	0.236	−0.043	0.507	0.091	0.035	0.912	0.178	−0.325	**0.007**	0.008	0.049	0.600

**Low 15-feet walking test (Slowness)**																
Unadjusted	0.288	1.334	[1.134, 1.570]	**<0.001**	0.112	0.523	**<0.001**	0.059	1.695	**0.008**	0.533	1.978	**<0.001**	0.059	−0.471	**0.008**
Adjusted*	0.275	1.316	[1.076, 1.609]	**<0.001**	0.252	0.244	0.098	0.108	0.201	0.516	0.531	1.982	**<0.001**	0.046	−0.444	**0.034**

**Self-reported weariness (Exhaustion)**																
Unadjusted	−1.405	0.245	[0.106, 0.568]	**0.001**	0.101	−3.631	**<0.001**	0.021	−7.353	0.120	0.141	−7.474	**<0.001**	<0.001	0.209	0.874
Adjusted*	−1.487	0.226	[0.078, 0.659]	**<0.001**	0.293	−1.551	0.133	0.891	0.289	0.349	0.210	−6.442	**0.001**	0.007	−0.559	0.704

**Unintentional reported (Weight loss)**																
Unadjusted	−0.706	0.493	[0.167, 1.458]	0.184	0.025	−2.354	0.086	<0.001	0.762	0.902	<0.001	0.135	0.955	0.021	−2.712	0.113
Adjusted*	−1.707	0.181	[0.044, 0.749]	**<0.001**	0.238	−1.134	0.379	0.095	0.279	0.363	0.122	−0.684	0.776	0.057	−4.379	**0.016**

**IPAQ - short version (Low PA levels)**																
Unadjusted	−0.460	0.632	[0.434, 0.919]	**0.013**	0.107	−1.754	**<0.001**	<0.001	0.043	0.985	0.176	−3.909	**<0.001**	0.031	1.173	**0.047**
Adjusted*	−0.245	0.783	[0.509,1.204]	**<0.001**	0.277	−1.182	**0.010**	0.094	0.270	0.382	0.253	−3.528	**<0.001**	0.029	1.053	0.110

* Adjusted for age, education level, morbidity index, body mass index and cognitive status (model 1). For each logistic regression and each of the FS components, the corresponding β coefficient, odds-ratio (OR), 95% confidence intervals for the OR and the p-value of the omnibus tests of model coefficients were computed. For each linear regression and each of the frailty components variables, the coefficient of determination of the model (R^2^), the β coefficient, the corresponding p-value and the p-value for the ANOVA test were computed; IPAQ = International Physical Activity Questionnaire
