# Supplementary material for: The Role of Physical Frailty Independent Components on Increased Disabilities in Institutionalized Older Women
Source: Transl Med UniSa. 2019 Jan 6;19:17–26. (PMC6581498)
Supplement: Supplementary file 1 [file TM-19-017-s001.doc]

| **Table 1.** Characterization of total sample and comparison of physical frailty subgroups for biosocial, global health and functional disability outcomes | | | | | |
| --- | --- | --- | --- | --- | --- |
|  | Total sample  (n=319, 100%) | Nonfrail  (n=49, 16%) | Pre-frail  (n=124, 38.7%) | Frail  (n=146, 45.4%) | p  value |
| **Sociodemographic** |  |  |  |  |  |
| Chronological age (years, A±SD) | 81,96 (±7,89) | 81.68 (±6.72) | 81.80 (± 8.65) | 82.19 (± 7.72) | 0.959 |
| Level of education (degree; M1;3) | 3 (3 ; 4) | 4 (3 ; 6) | 3 (3 ; 4) | 3 (2 ; 4) | 0.063 |
| Marital state (n,%) |  |  |  |  |  |
| Single | 31 (26.1) | 6 (31.6) | 12 (26.1) | 13 (24.1) |  |
| Married | 7 (5.9) | 4 (21.0) | 1 (2.2) | 2 (3.7) | 0.073 |
| Widowed or divorced | 81 (68.0) | 9 (47.4) | 33 (71.7) | 39 (72.2) |  |
| **Anthropometric data** |  |  |  |  |  |
| Weight (kilograms, A±SD) | 65.45(±12.58) | 66.22 (± 11.33) | 65.08 (±11.54) | 65.49 (± 13.98) | 0.946 |
| Stature (meters, M1;3) | 1.51 (1.47; 1.56) | 1.56 (1.49; 1.62) | 1.51 (1.47; 1.55) | 1.50 (1.46; 1.52) | **0.008** |
| Body mass index (A±SD) | 28.49 (± 5.05) | 26.95 (± 3.78) | 28.22 (± 4.60) | 29.27 (± 5.69) | 0.205 |
| **Clinical-mental health state** |  |  |  |  |  |
| Mini mental state (0-30 pts, M1;3) | 20 (15; 25) | 25 (21 ; 27) | 21 (17 ; 25) | 17 (13; 22) | **< 0.001** |
| Comorbidity index (0-10 pts, M1;3) | 7 (6; 9) | 8 (6 ; 10) | 7 (6 ; 8) | 8 (7; 9) | **0.026** |
| CES-D depression scale (0-60 pts, A±SD) | 21.92 (± 8.00) | 19.42 (± 7.99) | 19.46 (± 8.09) | 24.89 (± 6.98) | **0.001** |
| **Functional Disabilities indicators** |  |  |  |  |  |
| Katz index of ADL (0-6 pts, n,%; no disability) | 43 (36.1) | 13 (68.4) | 20 (43.5) | 10 (18.5) | **< 0.001** |
| Katz index of ADL (0-6 pts, n,%; no disability) | 76 (63.9) | 6 (31.6) | 26 (56.5) | 44 (81.5) |
| Lawton index of IADL index (9-32 pts A±SD) | 20.11 (± 5.70) | 17.37 (± 7.24) | 18.70 (± 5.27) | 22.28 (± 4.65) | **0.002** |
| Falls efficacy scale (10-100 pts M1;3) | 40.00 (18.00; 61.00) | 33.00 (14.00; 40.00) | 34.50 (13; 70) | 41.00 (26.00; 59.00) | **0.048** |
| Static balance test (per time, seconds M1;3) | 1.30 (0.05; 4.11) | 2.52 (0.71 ; 11.00) | 1.56 (0.17 ; 4.15) | 1.09 (0.01; 3.38) | **0.039** |
| Dynamic balance test (per time, seconds M1;3) | 13.00 (10.00; 20.56) | 9.75 (7.12; 10.58) | 11.15 (9.20 ; 14.90) | 20.14 (14.30; 25.97) | **< 0.001** |
| A=Average (mean), SD=standard deviation, M1; 3= Median (25^th^ Percentile; 75^th^ Percentile); pts = points | | | | | |
