# Supplementary material for: The Role of Physical Frailty Independent Components on Increased Disabilities in Institutionalized Older Women
Source: Transl Med UniSa. 2019 Jan 6;19:17–26. (PMC6581498)
Supplement: Supplementary file 2 [file TM-19-017-s002.doc]

**Table 2.** Characterization of total sample and comparison of physical frailty subgroups for functional disability outcomes

|  | 1 | 2 | 3 | 4 | 5 |
| --- | --- | --- | --- | --- | --- |
| 1. ADL |  |  |  |  |  |
| 2. IADL | **0.513**** |  |  |  |  |
|  | **0.472**** |  |  |  |  |
| 3. FES | 0.176 | **0.436**** |  |  |  |
|  | 0.114 | **0.329**** |  |  |  |
| 4. DBT | **0.428**** | **0.387**** | **0.321**** |  |  |
|  | **0.347**** | **0.261**** | **0.231*** |  |  |
| 5. SBT | **-0.255**** | **-0.299**** | **-0.253*** | **-0.466**** |  |
|  | **-0.240**** | **-0.261**** | **-0.227*** | **-0.449**** |  |
| 6. PF | **0.420**** | **0.327**** | **0.247**** | **0.662**** | **-0.224*** |
|  | **0.303**** | 0.149 | 0.140 | **0.610**** | **-0.194** |
| Notes: *p< 0.05 and **p< 0.010; in each variable line are expressed r and (p) values; partial correlation values are expressed in underline of each variable line and was adjusted for pre-determined covariates; PF =Physical frailty total score; ADL = Katz index; IADL = Lawton index; DBT = Dynamic balance test; SBT= static balance test. | | | | | |
