# Supplementary material for: The Role of Physical Frailty Independent Components on Increased Disabilities in Institutionalized Older Women
Source: Transl Med UniSa. 2019 Jan 6;19:17–26. (PMC6581498)
Supplement: Supplementary file 3 [file TM-19-017-s003.doc]

| **Table 3 –** Association between each physical frailty and functional disability components (n = 319) | | | | | | | | | | | | | | | | |
| --- | --- | --- | --- | --- | --- | --- | --- | --- | --- | --- | --- | --- | --- | --- | --- | --- |
| **Functional Disability outcomes** | **Katz’s index of ADL** | | | | **Lawton’s index of IADL** | | | **Falls efficacy scale** | | | **Agility-dynamic balance test** | | | **Static Balance test** | | |
| **Physical Frailty components** | β  coefficient | OR | 95% CI  for OR | omnibus  test  p-value | R^2^ | β  coefficient | p  value | R^2^ | β  coefficient | p  value | R^2^ | β  coefficient | p  value | R^2^ | β  coefficient | p  value |
| **Low hand grip strength test**  **(Weakness)** |  |  |  |  |  |  |  |  |  |  |  |  |  |  |  |  |
| Unadjusted | -0.056 | 0.946 | [0.898, 0.996] | **0.028** | 0.016 | -0.096 | 0.166 | <0.001 | 0.069 | 0.825 | 0.095 | -0.400 | **0.001** | 0.006 | 0.070 | 0.415 |
| Adjusted* | -0.047 | 0.954 | [0.896, 1.016] | **<0.001** | 0.236 | -0.043 | 0.507 | 0.091 | 0.035 | 0.912 | 0.178 | -0.325 | **0.007** | 0.008 | 0.049 | 0.600 |
| **Low 15-feet walking test**  **(Slowness)** |  |  |  |  |  |  |  |  |  |  |  |  |  |  |  |  |
| Unadjusted | 0.288 | 1.334 | [1.134, 1.570] | **<0.001** | 0.112 | 0.523 | **<0.001** | 0.059 | 1.695 | **0.008** | 0.533 | 1.978 | **<0.001** | 0.059 | -0.471 | **0.008** |
| Adjusted* | 0.275 | 1.316 | [1.076, 1.609] | **<0.001** | 0.252 | 0.244 | 0.098 | 0.108 | 0.201 | 0.516 | 0.531 | 1.982 | **<0.001** | 0.046 | -0.444 | **0.034** |
| **Self-reported weariness**  **(Exhaustion)** |  |  |  |  |  |  |  |  |  |  |  |  |  |  |  |  |
| Unadjusted | -1.405 | 0.245 | [0.106, 0.568] | **0.001** | 0.101 | -3.631 | **<0.001** | 0.021 | -7.353 | 0.120 | 0.141 | -7.474 | **<0.001** | <0.001 | 0.209 | 0.874 |
| Adjusted* | -1.487 | 0.226 | [0.078, 0.659] | **<0.001** | 0.293 | -1.551 | 0.133 | 0.891 | 0.289 | 0.349 | 0.210 | -6.442 | **0.001** | 0.007 | -0.559 | 0.704 |
| **Unintentional reported**  **(Weight loss)** |  |  |  |  |  |  |  |  |  |  |  |  |  |  |  |  |
| Unadjusted | -0.706 | 0.493 | [0.167, 1.458] | 0.184 | 0.025 | -2.354 | 0.086 | <0.001 | 0.762 | 0.902 | <0.001 | 0.135 | 0.955 | 0.021 | -2.712 | 0.113 |
| Adjusted* | -1.707 | 0.181 | [0.044, 0.749] | **<0.001** | 0.238 | -1.134 | 0.379 | 0.095 | 0.279 | 0.363 | 0.122 | -0.684 | 0.776 | 0.057 | -4.379 | **0.016** |
| **IPAQ - short version**  **(Low PA levels)** |  |  |  |  |  |  |  |  |  |  |  |  |  |  |  |  |
| Unadjusted | -0.460 | 0.632 | [0.434, 0.919] | **0.013** | 0.107 | -1.754 | **<0.001** | <0.001 | 0.043 | 0.985 | 0.176 | -3.909 | **<0.001** | 0.031 | 1.173 | **0.047** |
| Adjusted* | -0.245 | 0.783 | [0.509,1.204] | **<0.001** | 0.277 | -1.182 | **0.010** | 0.094 | 0.270 | 0.382 | 0.253 | -3.528 | **<0.001** | 0.029 | 1.053 | 0.110 |
| *Adjusted for age, education level, morbidity index, body mass index and cognitive status (model 1). For each logistic regression and each of the FS components, the corresponding β coefficient, odds-ratio (OR), 95% confidence intervals for the OR and the p-value of the omnibus tests of model coefficients were computed. For each linear regression and each of the frailty components variables, the coefficient of determination of the model (R^2^), the β coefficient, the corresponding p-value and the p-value for the ANOVA test were computed; IPAQ = International Physical Activity Questionnaire | | | | | | | | | | | | | | | | |
